# Does inhibin-B help us to confidently refuse diagnostic testicular biopsy in azoospermia?

**Published:** 2012-05

**Authors:** Mahmoudreza Moradi, Mohsen Alemi, Asaad Moradi, Babak Izadi, Farajollah Parhodah, Fatemeh Torkaman Asadi

**Affiliations:** 1 *Infertility Research Center, Urology-Nephrology Research Center, Kermanshah University of Medical Sciences, Kermanshah, Iran.*; 2 *Department of Pathology, Kermanshah University of Medical Sciences, Kermanshah, Iran.*; 3 *Department of Infectious Diseases, Hamedan University of Medical Sciences, Hamedan, Iran.*

**Keywords:** *Infertility*, *Histology*, *Sperm cell*, *Germ cell*, *Diagnosis*, *Azoospermia*.

## Abstract

**Background:** In the recent years, the use of laboratory blood factors such as FSH and inhibin-B for the assessment of spermatogenesis in different studies has increased; of course, the conflicting results have also been achieved.

**Objective:** To investigate if the measurement of inhibin-B can help surgeon to reduce unnecessary diagnostic testicular biopsies in males with azoospermia.

**Materials and Methods:** This cross-sectional study was done during July 2006 to September 2007 on 41 patients with azoospermia. FSH and inhibin-B were measured and bilateral open testicular biopsy was performed for all patients.

**Results:** Sperm was seen in 29% of biopsies that in 100% of these samples inhibin-B was more than 100 pg/mL and FSH was less than twice the normal (p=0.001). Inhibin-B had significant correlation inversely with testicular fibrosis and Sertoli cell only syndrome (p=0.043 and p=0.011, respectively) and directly with incomplete spermatocytic maturation arrest and obstructive azoospermia (p=0.027 and p=0.013, respectively). FSH was only correlated with obstructive azoospermia (p=0.001).

**Conclusion:** We suggest that if FSH is less than twice the normal, inhibin-B should be measured and if its level is less than 100 pg/mL, we can cancel about the half of unnecessary diagnostic testicular biopsies.

## Introduction

Male factors, alone or alongside female causes, can be involved in about 50% of infertile couples (1); therefore, diagnosis and treatment of its causes are particularly important. In the infertile patients with azoospermia, distinguishing obstructive causes from primary testicular failure is vital, and it will affect the treatment (2). A way to differentiate causes of obstructive from nonobstructive azoospermia is testicular biopsy with laboratory tests. There are possible complications of testicular biopsy such as; hematoma, wound infection, scrotal swelling and reducing the effective volume of the testis. 

Considering these complications plus the time and cost of testicular biopsy, its success rate can be predicted with the help of laboratory tests before the testicular biopsy, to reduce unnecessary performing of this invasive procedure. In the recent years, the use of laboratory blood factors such as FSH and inhibin-B for the assessment of spermatogenesis in different studies has increased; of course, the conflicting results have also been achieved and currently the primary measurement of inhibin-B isn’t satisfactory in the authentic urologic guidelines (3-5). FSH is a pituitary hormone that supports spermatogenesis by stimulating Sertoli cells in the seminiferous epithelium, and its secretion is controlled with the hypothalamic-pituitary-testis axis (6).

Inhibin-B is a glycoprotein hormone secreted by Sertoli cells, and suppresses FSH secretion by gonadotropes (7). The clinical use of inhibin-B in various studies as a marker of testicular function has been controversial (8). While some proposed that inhibin-B is an independent predictor of the presence of spermatozoa within the testis, or reported that inhibin-B levels are more sensitive than FSH level (9-13); others believed that both inhibin-B and FSH have been predictors of the presence of sperm in the testes of infertile men (14-17). 

Different studies have shown no difference between inhibin-B and FSH in the prediction of spermatogenesis (18-22). According to many controversies in various studies, we aimed to evaluate the correlation of serum FSH and inhibin-B with testicular histopathology and clear that when the measurement of inhibin-B can help surgeon for reduce unnecessary diagnostic testicular biopsies to conduct him for other decisions.

## Materials and methods

This cross-sectional study was done during July 2006 to September 2007 included 55 infertile patients referred to 4^th^ Shahid Mehrab Hospital of Kermanshah University of Medical Sciences with the primary diagnosis of azoospermia. This study was approved by the Ethic Committee of Kermanshah University of Medical Sciences. In all patients, detailed history, physical examination, hormone profile including testosterone, FSH, inhibin-B and Semen analysis were performed. Testicular volume was determined by ultrasonography. Then, those patients who had the criteria for inclusion in the study were selected and undergone bilateral open testicular biopsy. 

Inclusion criteria were azoospermia in two samples of semen analysis, testicular volume more than 10 ml, normal serum testosterone and obtaining informed consent from all subjects. Exclusion criteria were Vas deferens and epididymis disorders, secondary hypogonadism and severe atrophy of testis (volume less than 10 mL) 41 patients were selected to determine serum levels of synchronic FSH and inhibin-B and performance of biopsy. Patients underwent bilateral open biopsies of the testes, and the samples were evaluated by a skilled pathologist to determine the histopathology. Serum FSH using IRMA (IM2125 Immunotech kit, USA) and inhibin-B using ELISA (DSL-10-84100 kit, USA) were measured in a laboratory. 

**Statistical analysis**

The data were processed with SPSS version 16.0 (SPSS Inc., Chicago, IL). To compare continuous variables, Mann-Whitney U test were applied. P<0.05 was considered a statistically significant value.

## Results

The mean age of patients was 30.72±6.32 years (18-49 yrs). The mean infertility duration was 5.55±4.96 years (1.5-20 yrs). The mean serum FSH was 23.35±21.35 (2.70-95.50) ng/mL (normal value: 2.2-11 ng/mL). The mean serum levels of inhibin-B were 107.85±90.99 (2.00-383.30) pg/mL (normal value: >100pg/mL). The results of Histopathology of testicular biopsy were the following consecutively: Sertoli cell only syndrome (SCOS) 34.15% (n=14), Spermatogenesis maturation arrest (SMA) 26.83% (n=11) included 6 cases of Complete SMA and 5 Incomplete SMA. Testicular fibrosis (TF) 21.95% (n=9) and Obstructive azoospermia (OAZ) or normal spermatogenesis 17.07% (n=7).

The summary results of testicular histopathology and its relationship with FSH and inhibin-B values are shown in the table I and table II. Testicular volume was less than 15 ml in 56.1% (n=23) that 41.46% (n=17) were bilateral, and the remaining cases were unilateral. In this study, testicular volume with inhibin-B level had a direct correlation, but it was not significant (p=0.57), while serum FSH correlated with the volume inversely and significantly (p=0.001). 

In testes with histopathology of fibrosis (TF), serum inhibin-B level was less than 100 pg/mL in 88.9% and less than 200 pg/mL in 100% (p=0.043). Serum FSH level (in TF) was more than twice the normal in 66.7% and more than the normal in 100% (p=0.085). In testes with histopathology of Sertoli Cell Only Syndrome (SCOS), serum inhibin-B level was less than 100 pg/mL in 85.7% and less than 200 pg/mL in 92.8%, that it was statistically significant (p=0.011). However, serum FSH didn’t correlate with the SCOS (p=0.41).

In testes with histopathology of spermatocytic maturation arrest (SMA) serum inhibin-B level was less than 200 pg/mL in 81.8%, that it was 100% for complete SMA (p=0.057), while it was more than 150 pg/mL in 100% for incomplete SMA. Serum FSH level was normal in 64% for SMA (p=0.63), it was normal in 100% for incomplete SMA. In testes with normal histopathology (OAZ) serum inhibin-B level was more than 100 pg/mL in 100% (p=0.013) and serum FSH was normal in 85.7%, and less than twice the normal in 100% (p=0.001).

**Table I:** The correlation of serum inhibin-B and testicular histopathology

	**Inhibin-B (pg/mL)**				**p-value** *
	**1-100**	**101-200**	**201-300**	**301-400**	
Fibrosis % (n)	88.9 (8)	11.1 (1)	0 (0)	0 (0)	0.043
SCOS	85.7 (12)	7.15 (1)	7.15 (1)	0 (0)	0.011
SMA	27.3 (3)	54.5 (6)	18.2 (2)	0 (0)	0.06
OAZ	0 (0)	57.1 (4)	28.6 (2)	14.3 (1)	0.013

SCOS: Sertoli cell only syndrome, SMA: Spermatocytic maturation arrest, OAZ: Obstructive azoospermia.

* Mann-Whitney U test.

**Table II:** The correlation of serum FSH and testicular histopathology

	**FSH (ng/mL)**				**p-value** *
	**2.2-11**	**11.1-22**	**22.1-33**	**>33**	
Fibrosis % (n)	0 (0)	33.3 (3)	11.1 (1)	55.6 (5)	0.08
SCOS	14.3 (2)	35.7 (5)	35.7 (5)	14.3 (2)	0.41
SMA	63.6 (7)	9.1 (1)	9.1 (1)	18.2 (2)	0.63
OAZ	85.7 (6)	14.3 (1)	0 (0)	0 (0)	0.001

SCOS: Sertoli cell only syndrome, SMA: Spermatocytic maturation arrest, OAZ: Obstructive azoospermia.

* Mann-Whitney U test.

**Figure 1:**
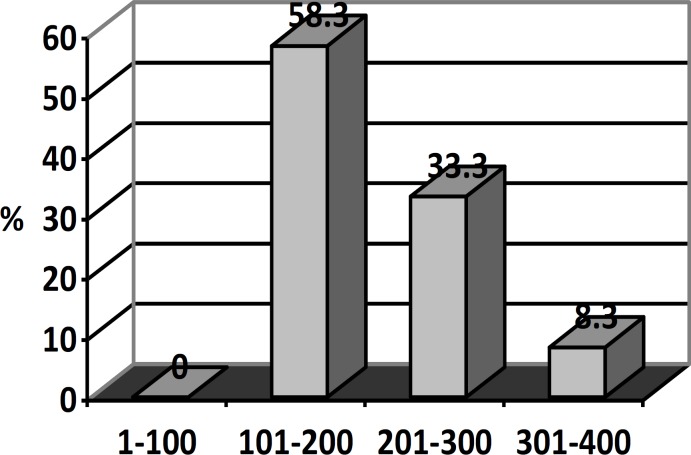
The sperm retrieval according to inhibin-B levels

**Figure 2:**
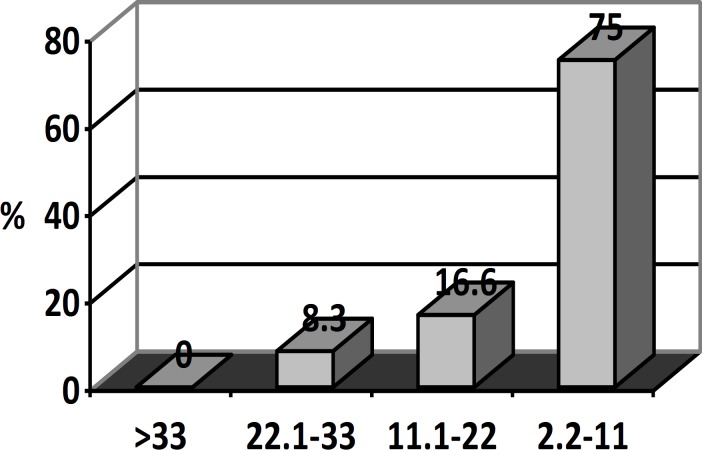
The sperm retrieval according to FSH level

## Discussion

In various studies, the conflicts that what factors are appropriate to predict the outcome of testicular biopsy are numerous. However, the role of FSH and inhibin-B in controlling hypothalamus-pituitary-gonad axis is undeniable (6-7). Therefore, using them to reach the proper diagnosis before aggressive procedures seems reasonable. Some studies believe that the value of inhibin-B in the prediction of spermatogenesis is higher than the value of FSH (9-13). Other studies (15, 18) asserted that the value of FSH is more. And some also defended this theory that these two tests are supplementary of each other (14, 16).

What we achieved in our study was that when inhibin-B is more than 150 pg/mL and FSH is less than twice the normal, the success rate of biopsy and sperm detection will be high, but these values didn’t differentiate obstructive azoospermia from others, especially from Incomplete SMA, because the hormonal changes in the obstructive azoospermia and Incomplete SMA are similar.

In our study, if inhibin-B was less than 100pg/mL and FSH was more than twice the normal, the success rate of biopsy was also low; so that 87% of patients with inhibin-B less than 100pg/mL and 81% with FSH more than twice the normal had histopathology of fibrosis or SCOS. Therefore, in such circumstances we can refuse biopsy; because the possibility of the presence of sperm would be less than 19%, especially if the testicular atrophy is present.

Ziaee and his colleagues used a combination of the inhibin-B and FSH in the prediction of testicular biopsy with positive predictive value 100%. Although they believed that their combination can be useful for decision making, it doesn’t cancel the need for biopsy so much (17). Nowroozi and his colleagues concluded that FSH can predict the presence of sperm, but inhibin-B cannot. They said that the combination of inhibin-B, FSH, and testes volume doesn’t take up the positive predictive value of biopsy (18). 

Von Eckardstein *et al* considered that inhibin-B sensitivity for prediction is a little more than FSH sensitivity and however, inhibin-B with FSH raises the sensitivity of prediction, but it cannot exactly predict the outcome of biopsy (16); that it conforms to our findings in the study.

Dimitrios *et al* allowed no preference for inhibin-B to FSH, and challenged that inhibin-B measurement before the biopsy is unnecessary (23). Smit concluded that inhibin-B cannot replace biopsy in suspected OAZ (24). Adamopoulos and his colleagues in a clinician's overview concluded that although inhibin-B and FSH are helpful in the detection of pathology of azoospermia, they are not superior to each other, and their role was not convincing in predicting biopsy (3).

The results of our study states that: even though the use of serum inhibin-B and FSH simultaneously for prediction of spermatogenesis is not accurate but can be helpful; so that if FSH is abnormal (more than twice) or inhibin-B is less than 100 pg/mL, the possibility of the sperm presence will be very little (in our study: 0%); so there is no indication for diagnostic biopsy. If FSH is less than twice the normal and inhibin-B is more than 150 pg/mL, there will be the possibility of both obstructive and Incomplete SMA pathology (29%); so in this situation, it seems that biopsy is reasonable and helpful to differentiate them. 

The main role of inhibin-B when is cleared that FSH is less than twice the normal; because according to our study 61% of azoospermic patients lie in this category and if inhibin-B is also less than 100pg/mL, then can be confidently refuse diagnostic biopsy and only about half of them remain to need testicular biopsy. The small sample was as one of our study limitation and it is necessary to more studies be performed in the future.

Currently, the measurement of inhibin-B in primary evaluation of azoospermic men isn’t recommended by guidelines but our data suggest that if FSH is less than twice the normal, the serum level of inhibin-B should be measured to cancel some of unnecessary diagnostic testicular biopsies, provided that its level will be less than 100 pg/mL.

## Conclusion

Based on the clinical criteria, serum FSH, and inhibin-B, the differentiation of azoospermia causes, especially obstructive azoospermia from incomplete SMA is not fully possible and biopsy would be the way decoder. Only in the cases that inhibin-B is less than 100pg/mL or FSH is more than twice the normal, because of the presence of testicular atrophy, we can confidently refuse diagnostic biopsy. Thus, if FSH is less than twice the normal, the measurement of inhibin-B is recommended

## Footnote

***This article extracted from residential thesis.***
